# Pigou in the 21st Century: a tribute on the occasion of the 100th anniversary of the publication of The Economics of Welfare

**DOI:** 10.1007/s10797-020-09653-y

**Published:** 2021-01-19

**Authors:** Ottmar Edenhofer, Max Franks, Matthias Kalkuhl

**Affiliations:** 1 Mercator Research Institute On Global Commons and Climate Change (MCC), Torgauer Str. 12-15, 10829 Berlin, Germany; 2 Technische Universität Berlin, Straße des 17, Juni 135, 10623 Berlin, Germany; 3 Potsdam Institute for Climate Impact Research, Telegraphenberg A 31, 14473 Potsdam, Germany; 4 Faculty of Economic and Social Sciences, University of Potsdam, August-Bebel-Straße 89, 14482 Potsdam, Germany

**Keywords:** Environmental economics, Climate change economics, Carbon pricing, Pigouvian taxation, Economic policy, Q5, H2

## Abstract

The year 2020 marks the centennial of the publication of Arthur Cecil Pigou’s magnum opus *The Economics of Welfare*. Pigou’s pricing principles have had an enduring influence on the academic debate, with a widespread consensus having emerged among economists that Pigouvian taxes or subsidies are theoretically desirable, but politically infeasible. In this article, we revisit Pigou’s contribution and argue that this consensus is somewhat spurious, particularly in two ways: (1) Economists are too quick to ignore the theoretical problems and subtleties that Pigouvian pricing still faces; (2) The wholesale skepticism concerning the political viability of Pigouvian pricing is at odds with its recent practical achievements. These two points are made by, first, outlining the theoretical and political challenges that include uncertainty about the social cost of carbon, the unclear relationship between the cost–benefit and cost-effectiveness approaches, distributional concerns, fragmented ministerial responsibilities, an unstable tax base, commitment problems, lack of acceptance and trust between government and citizens as well as incomplete international cooperation. Secondly, we discuss the recent political success of Pigouvian pricing, as evidenced by the German government’s 2019 climate policy reform and the EU’s Green Deal. We conclude by presenting a research agenda for addressing the remaining barriers that need to be overcome to make Pigouvian pricing a common political practice.

No “invisible hand” can be relied on to produce a good arrangement of the whole from a combination of separate treatments of the parts. It is therefore necessary that an authority of wider reach should intervene to tackle the collective problems of beauty, of air and light, as those other collective problems of gas and water have been tackled.

(Pigou 1920, Part I, Chapter VI, §11, pp. 170–171)

## Introduction

2020 marks the centennial of the publication of Arthur Cecil Pigou’s magnum opus *The Economics of Welfare*. In this masterpiece of economic theory, Pigou set out “to study certain important groups of causes affecting economic welfare in actual modern societies” (Pigou 1920, Part I, Chapter I, §5, p. 11). It is noteworthy that Pigou used the term “actual modern societies” when stating the objective of his mission. Over the course of his early career leading up to the publication of his magnum opus, Pigou became a disciple of Alfred Marshall. As such, he supported Marshall’s efforts to establish economics as a discipline in its own right (Maloney 1985), setting it apart from philosophy and history. In particular, he distanced himself from economic historians, who, since the *Methodenstreit* in the 1880s, saw themselves as being increasingly separated from the new orthodoxy in economics, which now studied neo-classical equilibria and marginal utility (Hobsbawm 1987, chap. 11).

Pigou was heavily engaged in contemporary politics and public debates of his times. In several columns, penned by the young Pigou, he advocated free-trade principles and argued against Chamberlain’s protectionist trade policies. Reacting to a senior cabinet member’s campaign, he mounted a defense of pension schemes that retain incentives to work. His writings on the efficiency of land rent taxation engendered a heated controversy among liberal members of parliament. Pigou continued working on social questions, such as minimum housing standards, unemployment benefits and medical insurance (Takami 2014).

In this tribute, however, we wish to focus on Pigou’s contributions that laid the groundwork for the modern field of environmental economics (Sandmo 2015). In *The Economics of Welfare*, Pigou defined positive and negative externalities, described their impacts on production and devised ways of correcting or internalizing these externalities:

In like manner, for every industry in which the value of the marginal social net product is less than that of the marginal private net product, there will be certain rates of tax, the imposition of which by the State would increase the size of the national dividend and increase economic welfare; and one rate of tax, which would have the optimum effect in this respect.

(Pigou 1932, 4th ed., Part II, Chapter XI, §11)

Local air pollution was already a significant problem at that time, as dust led to higher electricity demand (lightening) as well as higher costs for cleaning buildings and clothes—a problem that Pigou highlights in his book by citing a governmental report^1^:

A valuable investigation was made in 1918 by the Manchester Air Pollution Advisory Board into the comparative cost of household washing in Manchester—a smoky town—as compared with Harrogate—a clean town. The investigator obtained 100 properly comparable statements for Manchester and Harrogate respectively as to the cost of the weekly washing in working-class houses. These showed an extra cost in Manchester of 7½d. a week per household for fuel and washing material. The total loss for the whole city, taking the extra cost of fuel and washing materials alone, disregarding the extra labor involved, and assuming no greater loss for middle-class than for working-class households (a considerable under-statement), works out at over £290,000 a year for a population of three quarters of a million.

(Pigou 1932, 4th ed. Part II, Chapter IX, §10, Footnote 68)

Today, we see Pigou’s legacy in the fundamental concepts of externalities and their correction through Pigouvian taxes or subsidies, which are taught in elementary economics courses. Modern research on optimal environmental taxation has often equated a tax set at marginal environmental damages with the Pigouvian tax (Cremer et al. 1998). In this line, various works have analyzed optimal policies in so-called second-best settings, that is, when there exist market failures or distortions besides the environmental externality. Examples are different forms of heterogeneity combined with imperfect information, leakage effects due to jurisdictional spillovers, market concentration, innovation market failures, etc. In these settings, the fundamental research question is often whether the optimal environmental tax deviates from the ‘Pigouvian level’—and in what direction (Aronsson and Sjögren 2018; Cremer et al. 2003; Jacobs and De Mooij 2015; Jacobs and van der Ploeg 2019; Parry 1995; Requate 2005a, 2005b; Van der Ploeg 2016).

These findings confirm, rather than disprove, the Pigouvian idea, as the underlying principle corresponds to Pigou’s approach of setting a tax that maximizes welfare. In that sense, Pigou’s claim that “there will be certain rates of tax [that] would increase […] economic welfare; and one rate of tax, which would have the optimum effect in this respect” also holds in second-best settings. Hence, the widespread convention of equating the ‘Pigouvian tax’ with marginal environmental damages, rather than the optimal second-best tax rate, fails to do justice to Pigou’s work. In what follows, we, therefore, consider a broader definition of Pigouvian pricing policies, namely those that maximize welfare (in either first-best or second-best settings).

Notwithstanding the strong economic argument for Pigouvian pricing, environmental policy in many countries has been dominated by regulation, standards, and, in some cases, emission trading schemes (Cullenward and Victor 2020; Keohane et al. 1997). Despite strong support for stricter environmental policy, we see little public support for using environmental taxes (European Commission 2019)—neither for estimated second-best tax rates, nor for estimated first-best Pigouvian taxes.

**Fig. 1:**
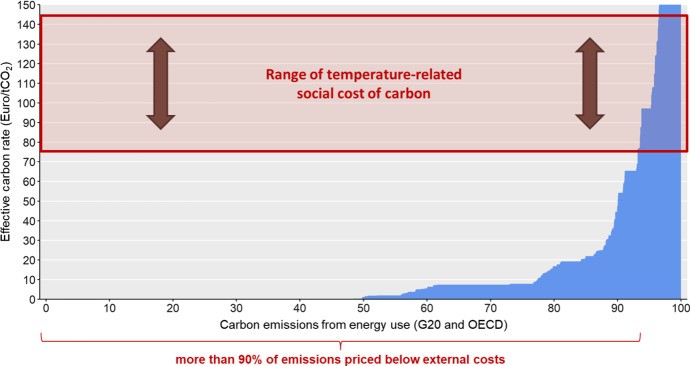
Effective carbon prices resulting from existing excise taxes, duties and carbon prices on fossil energy. Source: own figure based on OECD’s effective carbon rate data in Kalkuhl et al. (2018) and temperature-related social costs of carbon (Kalkuhl and Wenz, 2020). Note that subsidies on fossil fuels are not included in this figure

There are only few examples of Pigouvian taxes that attempt to reflect marginal social benefits—the US oil extraction tax to pay a fund for cleaning up oil pollution accidents is a rare example, where the tax rate is determined by a social cost consideration (Masur and Posner 2015). Even if taxes or levies are set to reduce environmental pollution, they are often based on political considerations rather than on rigorous assessments of the marginal social cost. Hence, many taxes that are part of the environmental policy mix, if they can be called Pigouvian at all, do not reflect the true marginal social costs—but the balance of political power of different interest groups. Similarly, energy and fuel taxes are often below their Pigouvian optimum (Coady et al. 2018) when taking into account the externalities associated with global warming or local air pollution. Globally, most taxes on fossil energy (irrespective of whether they are explicitly targeted at carbon emissions or not) are still far below the social cost of carbon (see Fig. 1). In some cases, the effective carbon price is even negative due to subsidies on fossil fuels (not shown in Fig. 1 due to data reasons). The *International Energy Agency* estimates that annual global expenditures for subsidies on fossil fuels averaged approximately US$ 340 billion in the period from 2016 to 2019.^2^ Similar to fuel taxes, taxes on tobacco and alcohol are often set based on revenue motives (Masur and Posner 2015). While some countries, like the US and the UK, require regulatory impact assessments of social costs and benefits of policy proposals, the latter have rarely resulted in taxes that conform to Pigouvian pricing principles. Instead, these assessments, more often than not, merely rationalize other environmental policies, such as fuel efficiency standards.

Pigou’s work is still of utmost relevance today, especially since the chasm between the theory of Pigouvian pricing and its political implementation remains significant, as the above examples illustrate. Large market failures remain unaddressed—among them the adverse effects of greenhouse gas emissions, the loss of biodiversity and the under-provision of public goods related to innovation, knowledge and health services (e.g., vaccination).

In this article, we will revisit the relevance of the Pigouvian pricing principle for contemporary economic research and policy-making by defending two basic claims:

1. While economists are right to believe Pigouvian taxes to be theoretically desirable, they are also prone to overlooking the significant theoretical problems that Pigouvian taxes still face. Paying scant attention to these conceptual problems is, however, likely to impede their implementation in practice.
2. The widely held belief that Pigouvian taxes are ‘politically infeasible’ rests on a rather flimsy factual basis—as is borne out by an increasing number of countries adopting carbon prices.

These two claims imply that the widespread consensus, namely that Pigouvian taxes are theoretically desirable, yet politically infeasible, throws a spanner in the works of scientific progress. Specifically, holding on to this consensus impedes necessary research into conceptual problems, whilst inducing economists to abandon welfare-maximizing policies too readily, instead accepting politically less demanding options. Both tendencies cause additional deadweight losses to societies.

We will focus on the case of climate change due to the ongoing political ambitions to limit greenhouse gas (GHG) emissions in many countries and the lessons learned from successful and failed attempts to price carbon. We will use the term Pigouvian taxation to refer to direct pricing policies, such as taxes and levies—as opposed to indirect pricing via emissions certificate trading. A price collar is subsumed under direct pricing.

The remainder of this article is structured as follows: We outline key conceptual challenges for implementing Pigouvian taxes (Sect. 2), we then discuss how current climate policy has increasingly moved toward Pigouvian principles and carbon pricing (Sect. 3), and finally suggest a research agenda (Sect. 4) for addressing remaining barriers to make Pigouvian pricing a common political practice. We conclude (Sect. 5) with some reflections on the legacy of Pigou’s work for contemporary economic policy.

## Barriers to implementing Pigouvian taxes and subsidies

There is a broad consensus on the theoretical optimality of Pigouvian taxes among a diverse set of economic, political and philosophical thinkers.^3^ However, implementing Pigouvian taxes and subsidies is fraught with several theoretical and political challenges that have often impeded their use in favor of other regulatory measures. In the following, we will briefly sketch these challenges.

### Uncertainties about marginal benefits, e.g., the social cost of carbon

A Pigouvian tax on carbon emissions has to be set equal to the social cost of carbon (SCC), which refers to the discounted marginal damages from a marginal increase in carbon emissions along a pre-defined emission path. Uncertainties about the social cost of carbon are considerable, with values from well-established integrated assessment models (IAMs) ranging from 7 US$/tCO_2_ in FUND (Waldhoff et al. 2011), to 31 US$/tCO_2_ in DICE (Nordhaus 2017) and much higher values exceeding 100 US$/tCO_2_ obtained from recent econometric analyses (e.g., Kalkuhl and Wenz 2020; Ricke et al. 2018) or expert surveys (Pindyck 2019). In addition to uncertainties with respect to the climate system, technologies or climate damages (Gillingham et al. 2018), the SCC strongly depends on normative choices regarding discount rates (Nordhaus 2017) and equity weights (Adler et al. 2017; Anthoff and Emmerling 2018).

### Unclear relation between cost–benefit analysis and cost-effectiveness analysis

Because of the large uncertainty range, the difficulties associated with quantifying catastrophic risk and non-market damages as well as the elusive search for agreement on fundamental normative parameters, the intergovernmental panel on climate change (IPCC) has not used the concept of the SCC for policy advice. It has, instead, focused on qualitative and quantitative impacts of warming for different temperature levels. Quantity targets on emissions, temperature targets or the time when ‘carbon neutrality’ is achieved are easier to communicate to the public—as these targets convey a very concrete environmental outcome. The environmental implication of a carbon price is, however, uncertain and unclear for many people. Politically, the uncertainty associated with the SCC and the relation between cost-effectiveness analysis (CEA) and cost–benefit analysis (CBA) has called the political viability of Pigouvian taxation into question, as the political focus is on reducing emissions rather than internalizing external costs. Accordingly, environmental targets play a more important role internationally and at the EU level.

From an economic perspective, any emission target can—in principle—be achieved by an emissions tax. The emissions tax is a cost efficient instrument for achieving the target, even if it is not optimal from a social welfare perspective (Baumol and Oates 1971). In this respect, a welfare-maximizing approach and a cost-efficiency approach may imply the same choice of the policy instruments, although tax levels—and the procedure of their adjustments—may differ.

Besides these seemingly subtle differences between a target-based (CEA) and a welfare-based (CBA) approach, it remains unclear for many economists how to reconcile the two concepts. Typically, studies a priori decide to follow either the one or the other approach. However, both approaches can inform and strengthen each other. A politically set climate target that deviates strongly from a welfare-maximizing climate target constitutes an inconsistency that should be resolved. The discrepancy reflects to some extent the limited possibilities to incorporate deep uncertainty, systemic risks or non-market damages into traditional cost–benefit modeling. As economists have started to incorporate these aspects into their models, optimal temperature targets have often decreased and the SCC increased, implying a narrowing gap between political targets and welfare-optimal targets (Dietz and Venmans 2019; Hänsel et al. 2020; Lemoine and Traeger 2014, 2016a, 2016b). Thus, the long-standing discrepancy has inspired economic works to account for various aspects of global warming that had previously been neglected. But politically set targets should also be adjusted to new insights from cost–benefit analyses. For example, cost–benefit analyses could help us identify the conditions and normative assumptions required for more stringent climate targets like the 1.5 °C target to be welfare-optimal.

The difference between target-based and welfare-optimization-based approaches diminishes if targets are understood as the outcome of a decision process that involves broader social cost–benefit considerations under uncertainty. Politically, this implies that climate targets are considered provisional goals, which provide orientation for the future development of our societies. Crucially, however, significant political capacity is required to adjust and update these targets with new scientific insights.

### Distributional concerns

Distributional concerns are at the heart of policy-making. Pigouvian pricing therefore faces the challenge of ensuring an equitable distribution of the burdens it creates among heterogeneous households as well as among heterogeneous firms.

Households choose consumption baskets with strongly varying carbon intensities. In industrialized countries, there is typically a negative correlation between income and carbon intensity, giving rise to a regressive effect of Pigouvian carbon pricing—an issue of vertical distribution. However, there is reason to believe that the heterogeneity in carbon intensity within one income group—an issue of horizontal equity (Pizer and Sexton 2019)—is politically and economically at least as important as the questions dealing with vertical equity. In public debates and the media, hardship cases of households with high carbon footprints (e.g., commuters living in badly insulated homes) are frequently used to criticize carbon pricing on account of the unfair distributional outcomes it engenders.

However, regressive distributional effects might turn out to be less severe when further general-equilibrium effects are taken into account explicitly. As long as the economy as a whole is relatively carbon-intensive, carbon taxes put a higher burden on capital income compared to wages because capital-intensive industries are more carbon-intensive (Goulder et al. 2019; Rausch et al. 2011).

Firms and investors, on the other hand, face the problem of stranded assets. The threat is particularly relevant for owners of fossil resources who stand to lose large rents (Bauer et al. 2016; Kalkuhl and Brecha 2013; van der Ploeg and Rezai 2020)—but also for ‘brown capital’ which cannot be converted into ‘green capital’ (Kalkuhl et al. 2020; Rozenberg et al. 2020). Moreover, the stability of the financial system could be compromised (Carney 2016).

As a Pigouvian tax constitutes a potential Pareto-improvement, there should theoretically be a way of redistributing the tax revenues that makes everyone better off. Such a redistribution scheme is, however, difficult to implement given the informational requirements, the transaction costs, potential incentive problems and the diffuse as well as uncertain nature of various environmental damages. Because of the direct and immediately felt costs of Pigouvian taxation, which contrast with the more abstract and aggregate benefits, policy-makers and the public have to take a leap of faith when putting their trust in the efficiency-enhancing impact.

The consideration of distributional effects is a prime example of Pigouvian taxes under second-best conditions. The early works on the double dividend hypothesis, for example, studied how environmental taxes interact with a distortionary tax system (Bovenberg and Goulder 1996; Parry and Bento 2000; Phaneuf and Requate 2017). This strand of research *assumed* that lump-sum taxes are impossible and public revenues *have to be raised* through distortionary labor taxes. A key question this literature has sought to address was whether the optimal environmental tax should be higher or lower than the marginal damages. Later works, however, have relaxed the assumption of infeasible lump-sum transfers and added an explicit distributional motive to the social welfare function, for example Cremer et al. (1998, 2003) and Jacobs and van der Ploeg (2019). This setting generates an endogenous (optimality) argument to rely on distortionary income taxes for financing the government budget. Under specific conditions, equity and efficiency can be separated (Aronsson and Sjögren 2018, provide an excellent overview). While the optimal environmental tax typically includes a component reflecting the marginal environmental damages, it may also contain further terms accounting for heterogeneity of households and overall costs of public funds.

### Fragmented responsibilities

The executive branch of a typical modern (democratic) government is divided into separate ministries. Since contemporary economies strongly depend on fossil fuels as an input, multiple ministries, apart from the ministry for the environment, are responsible for mitigating the emission of greenhouse gases (GHGs). The target-based approach mentioned above implies that national emission targets are split into sectoral targets with specific ministries responsible for achieving ‘their’ goals. There are ministries responsible for specific GHG emitting sectors of a national economy (transportation and infrastructure, agriculture, housing and building, industry and power) and ministries for cross-cutting areas (finance ministry, ministry of economics, foreign ministry). Due to the lack of a ministry for climate change mitigation, responsibilities are fragmented. The resulting lack of coordination has led to an excessive focus on sector-specific policies and technology policies. Consequently, most carbon pricing initiatives that exist today cover only a subset of all economic sectors. The European Emissions Trading Scheme (EU ETS), for example, covers only the power sector, industry and aviation. Agriculture, transport and the building sectors have been left out. Within these sectors, different ministries are responsible for reducing emissions. However, these ministries are targeted by the respective lobby groups. From a political economy perspective, ministries are likely to provide additional subsidies when emission targets are not fulfilled. As set out in a recent German government report, most of these subsidy schemes are ineffective in this context (Deutsche Bundesregierung 2020).

The sectoral approach implies substantial costs that arise in virtue of the reduced flexibility to avoid emissions where it is cheapest. Additionally, ignoring sectoral trade-offs, synergies and spillovers, leads to misallocation of mitigation efforts and investments. Harmonizing carbon prices across the EU-ETS, the transport and building sectors—that are not covered by the EU-ETS—could yield welfare gains of more than 1%, in particular in later periods when carbon price differentials will increase substantially (Hübler and Löschel 2013). There is further compelling theoretical and empirical evidence that misallocation across sectors increases not only static mitigation costs but also reduces total factor productivity (Banerjee and Moll 2010; Moll 2014). Implementing a carbon price therefore requires overcoming the ‘divide-and-conquer’ principle of many modern bureaucracies. However, it also implies a delegation of power as specific, previously used sectoral instruments become obsolete under uniform and harmonized carbon pricing. Until harmonized carbon pricing is achieved, sectoral policies will remain important, especially when aimed at compensating carbon-intensive industries or households, addressing complementary market failures and providing public goods, e.g., related to infrastructure, innovation and public investments.

### Unstable tax base

One fundamental objective of every government consists in ensuring that there are sufficient public funds to finance public goods. Pigouvian taxation, with the objective of internalizing the climate externality, would generate substantial public revenues with estimates ranging from 1 to 6% of national GDP (Jakob et al. 2016; Franks et al. 2018; Kalkuhl et al. 2018; IMF 2019a) and would thus be a part of the national fiscal system. For those governments that have pledged to achieve carbon neutrality by the middle of the twenty-first century the additional tax revenues associated with environmental Pigouvian taxes are only temporary. Moreover, it is hard to predict how fast a given carbon tax path will lead to the desired GHG emission reductions; it is even harder to predict the price evolution of emission certificates when, instead of a tax, an ETS is implemented. The upshot is that greenhouse gases are an unstable tax base, which makes the construction of a general system of public finances that is consistent with reducing emissions a non-trivial task for finance ministers.

Environmental ministers, on the other hand, are responsible for achieving the environmental goals of emissions reduction. There is a tendency for them to choose climate policy instruments under the constraint that costly coordination with the ministry of finance should be avoided. Quantity instruments, standards and trading schemes are thus preferred over carbon taxes. The problem of the unstable tax base can thus also be understood as a problem of fragmented responsibilities.

### Commitment problems

Climate policy requires a substantial shift in investments from brown to green technologies (IEA 2018; McCollum et al. 2018). Investments worth approximately 1% of current global GDP would have to be re-directed if we are to limit the rise in global mean temperature to 2 °C. Carbon prices can only trigger such enormous shifts in investments if they are highly credible, that is, if investors expect carbon prices to increase over time on a trajectory that is consistent with the 2 °C target. Governments have thus far lacked the ability to credibly commit themselves to such carbon price trajectories since they are subject to the vagaries and (dynamic) inconsistencies that result from electoral competition. Nor have they delegated the decision to set carbon price paths to an independent body. This creates political uncertainty, which undermines the dynamic efficiency of carbon pricing and slows investment shifts and innovation.

Besides political uncertainties, time inconsistency problems further aggravate the formation of stable expectations about announced future Pigouvian price paths. Governments are driven by various motives. These include fiscal motives, seeking specific rents from powerful economic groups and re-election motives. Each of these three reasons alone provides an incentive to deviate from announced carbon price paths, rendering government decisions time-inconsistent (Harstad 2019; Kalkuhl et al. 2020). Moreover, Pigouvian taxation for national purposes may be misused to either mimic tariffs or extract foreign rents (Amundsen and Schöb 1999; Franks et al. 2017).

The problem of commitment is less pronounced for standards or investment subsidies—which directly affect investment decisions—but imply higher costs in reducing emissions. Likewise, sectoral mitigation targets might help to foster expectations and commitment because they can increase the institutional incentives of ministries to achieve these targets. The preceding shows why policy-makers tend to have little trust that carbon prices induce dynamically efficient investment and innovation decisions. This is supported by the skeptical reasoning of environmentalists or non-economic scientists that carbon pricing is good at harvesting the currently low-hanging fruits to mitigate emissions, but may not enable plantation of fruit trees for future harvest (Rosenbloom et al. 2020). Without dynamically efficient and credible carbon prices, markets might actually favor abatement options that are dynamically inefficient (Vogt-Schilb et al. 2018).

### Lack of acceptance/commodification objection

Carbon pricing and carbon markets are perceived as repugnant by some environmental groups. Page (2011), for example, holds that the permit trading mechanism established in the Kyoto Protocol erodes norms of responsibility in the future, commodifies the atmosphere in an illegitimate manner and erodes the environmental morale. In the same vein, Sandel (2012), raises fundamental moral objections to market mechanisms. Moreover, in an experimental setting, Jakob et al. (2017) have shown that market-based policies, while efficient, conflict with moral behavior. According to the authors, moral responsibility induced study participants to take inefficient actions that reduced the earnings of the whole group of participants.

### Trust between governments and citizens

Klenert et al. (2018) argue that Pigouvian taxation is more acceptable to voters when the general level of trust between citizens and government is rather high. The recent protests of the yellow vest movement in France are a case in point, illustrating that high levels of distrust can lead to public opposition to Pigouvian taxation. Preliminary results of a recent survey-based study in France (Douenne and Fabre, 2020, SURED Conference) show that respondents who oppose the tax tend to discard positive information about it, which would be consistent with distrust, uncertainty, or motivated reasoning.

### Incomplete international cooperation

Finally, Pigouvian taxation could work well for national environmental problems where a national government exists. For transboundary problems, a regional or global government would be needed to implement Pigouvian taxes. In the absence of the latter, national governments are subject to free-rider problems. Even governments that are committed to reducing emissions are constrained by carbon leakage problems, when implementing high carbon prices. Empirically, carbon leakage has played only a very minor role so far (see, e.g., Naegele and Zaklan 2019). However, with rising carbon prices, leakage might become a more severe problem (Babiker 2005).

## Pigouvian pricing in the wild: a drama in three acts

Despite all the challenges associated with putting Pigouvian taxes into practice, carbon pricing, across the world, has increased considerably since the early 1990s when Finland and Sweden were the first countries to implement a carbon tax. According to the World Bank’s carbon pricing dashboard, as of the year 2020, 16% of global GHG emissions are subject to an explicit carbon price—typically, though, at relatively low levels.^4^ 6% of emissions are regulated under carbon taxes compared to 10% under emissions trading schemes, where emission targets are set and prices evolve endogenously.

In the following, we will focus on carbon pricing in actual modern societies. We will trace Pigou’s legacy to Germany’s recently enacted carbon price reform (Act 1), to the European Union’s Green Deal proposal (Act 2) and finally to reform proposals aimed at increasing cooperation at the international level (Act 3). As was and still is to be expected, the practical application of Pigouvian policies provides a rich set of lessons. Hence, at the end of this section, we will discuss how much progress has been made in overcoming the barriers that we have outlined in Sect. 2 and summarize the lessons learned and their implications for the future of carbon pricing. The implementation of carbon prices is—despite widespread skepticism—a real option for policy-makers.

### Act 1: Germany’s carbon price reform

Germany provides a prime example for a recent carbon price reform, introducing a paradigm shift in climate policy. The German case illustrates how quickly a pricing system can be implemented that was previously assessed ‘politically infeasible’ by many experts and economists. Policy-makers across the political spectrum as well as the general public were extremely wary, or outright distrustful, of using price signals to promote environmental goals. For decades, the German government applied regulation, command-and-control policies, subsidies and standards to reduce (fossil) energy consumption. In the early 2000s, the coalition government, consisting of the Social Democrats and the Greens, implemented an environmental tax reform that increased energy taxes, whilst shying away from implementing economy-wide, harmonized carbon prices, which would increase over time. Finally, in 2019, the German government initiated a consultation process between various ministries, experts and scientists to design a new climate policy package that would include, among several other measures, a national carbon price for the heating and transport sectors.

The drive to pass any meaningful climate legislation was partly due to pressure by the young generation. In particular, the *Fridays for Future* movement emerged from the center of society and also managed to make parts of the (conservative) establishment take their concerns seriously. Yet, substantial pressure also came from the EU Effort Sharing Regulation that mandates emission reductions in the non-EU-ETS sectors, in particular in the heating, transport and agriculture sectors.

Non-compliance with the EU Effort Sharing Regulation, which was adopted in 2018, entails substantial costs to the respective government. A non-compliant member state can only avoid penalty payments to the EU Commission if another member state manages to exceed its specific targets (by emitting less than originally intended) and sells its remaining quota to the non-compliant member state. The price of such remaining quotas is highly uncertain; it is not certain whether there will be any supply at all. In any case, the German government also feared a strong reputation loss should it violate EU norms and not meet its self-imposed emission target.

Early on, the government aimed at introducing a carbon price of some form to cover the heating and the transport sectors, while GHG emission reductions in the agriculture sector would be achieved by other means. The German debate centered around two design questions: whether to implement a price or a quantity instrument to establish a carbon price and how to use the revenues. The price vs. quantity debate divided the respective political camps, with market liberals and conservatives opting for a ‘marked-based’ ETS and Social Democrats favoring the ‘tax’.

The assessment by MCC and PIK, that is the *Mercator Research Institute on Global Commons and Climate Change* and the *Potsdam Institute for Climate Impact Research* (Edenhofer et al. 2019), in combination with a report by the Economic Advisory Council (Sachverständigenrat zur Begutachtung der gesamtwirtschaftlichen Entwicklung 2019), emphasized that both approaches can be designed in such a way that very similar outcomes are achieved. In particular, the carbon tax would require frequent adjustments if emission targets by 2030 are to be met with certainty. The MCC-PIK assessment, however, also discussed a hybrid model starting with a fixed-price ETS (which can be implemented rather quickly) that is then transformed into an ETS with a price collar, once the necessary regulatory steps—particularly the creation of infrastructure and auctioning processes—have been completed. Starting with a fixed-price ETS, rather than a temporary carbon tax, avoids potential legal problems. German constitutional law, in fact, might not have allowed the introduction of a direct tax on carbon emissions; rather, existing excise tax rates on various fossil energy types could be modified and harmonized according to their carbon content (Büdenbender 2019). The ETS with a price collar has two further advantages. It guarantees planning reliability for investments (minimum price) and simultaneously increases commitment and credibility of the ETS because costs cannot increase above the maximum price.

**Fig. 2:**
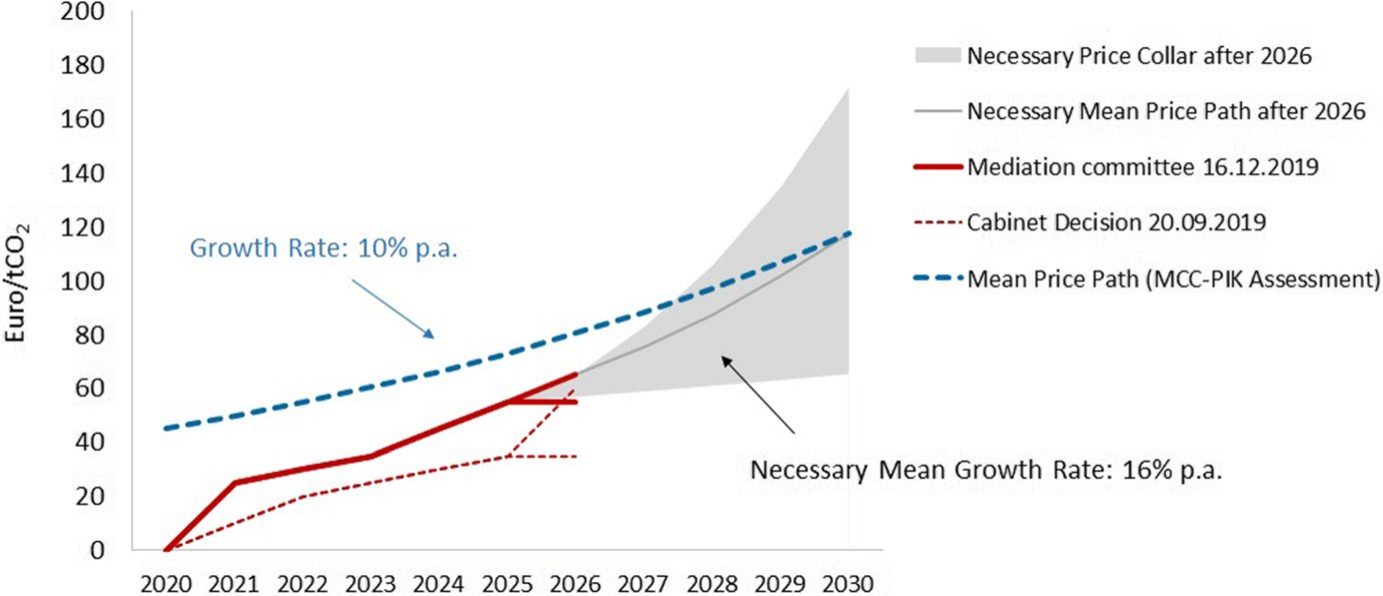
Carbon price for non-ETS sectors in Germany (own illustration, based on Edenhofer et al. 2020)

The government decided to implement the fixed ETS price starting at 10 €/tCO_2_ in 2021 that will then morph into an ETS with minimum and maximum prices. The specific levels of the carbon prices were the outcome of a political bargaining process among coalition parties. In the ensuing legislative process, the Greens, as an opposition party, used their leverage in the second chamber of parliament (Bundesrat) to increase carbon prices further. Ultimately, it was decided that the price will start at 25 €/tCO_2_ in 2021, increase to 55 €/tCO_2_ in 2025 and, after that, the price will be formed by the forces of supply and demand on the market for certificates, where the latter is supplemented with a price collar (Fig. 2). Still, prices are below the levels that are likely needed to achieve the reduction in emissions that the German government has committed itself to.

The revenues of the German national carbon price are expected to make up a significant share of total financing of the whole climate policy package. Together with the revenues generated by the EU ETS, carbon pricing is estimated to generate about 80% of the €62 billion that the government has allocated to public spending over the years 2020 to 2023 for the achievement of the emission reduction targets. The remaining 20% are financed from the general government budget. Most of the climate-related spending is allocated to programs to reduce emissions or energy use, e.g., in the building, industry or transport sectors. In spite of the strong emphasis on distributional concerns in public debates, only about 25% of total spending, that is about €15 billion over the next four years, will be used to address adverse distributional impacts (Knopf 2020).

**Fig. 3:**
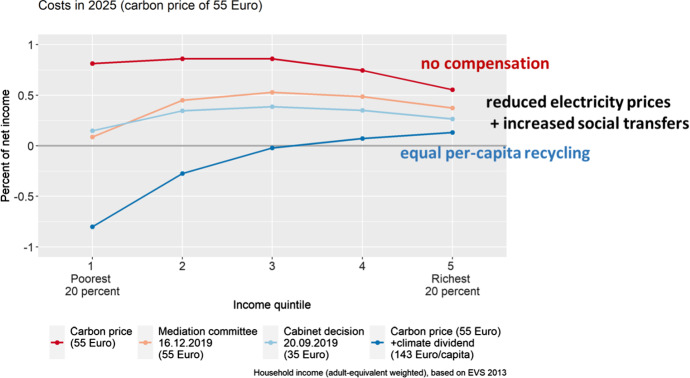
Distributional incidence of the carbon price in 2025. Own illustration, based on Edenhofer et al. (2020). Costs indicate real income losses due to increasing energy prices

Indeed, distributional concerns were at the center of the discourse leading up to the adoption of the carbon pricing reform. Several proposals were made for accompanying measures to compensate the losers of the reform, such as commuters and owners of badly insulated houses. The term “unsanierter Fernpendler” (long-distance commuters that live in badly insulated houses) was coined to describe the societal group that would be hit hardest by the reform. The debates around the reform and its distributional consequences revealed the importance of horizontal equity concerns (see also Fischer and Pizer 2019). The option of giving back revenues directly to citizens via a new transfer scheme was dismissed due to administrative hurdles and because some politicians questioned the effectiveness of carbon prices when revenues are completely recycled back to citizens.^5^ Instead, the government put together a bundle of alternative compensation measures. Of the €15 billion in that bundle, 80% (€12 billion) are used to reduce electricity prices by lowering the national renewable energy levy (EEG-Umlage), 7% (€1.1 billion) to increase direct and indirect transfers to long-distance commuters and 1% (€0.2 billion) to increase the housing allowance (Knopf 2020). Without any revenue recycling, the carbon price would have inflicted an income loss of approximately 0.8% on low- and middle-income households—while wealthier households would have faced only a 0.5% income loss (see Fig. 3). The estimates provide a first-order approximation of the distributional incidence of energy price increases, ignoring any supply- or demand-side responses. If all revenues from the national carbon price were redistributed on an equal-per-capita base (‘climate dividend’), low-income households would benefit considerably with middle-income households being hardly affected. Overall, with the compensation due to reduced energy prices and increased social transfers, the lowest income group is hardly affected at all, while the middle class bears the largest—yet still moderate—burden of the carbon price incidence. Interestingly, the increase in the 2025 carbon price from 35 €/tCO_2_ (cabinet decision) to 55 €/tCO_2_ (mediation committee between both legislative chambers) reduced the expected costs to the poorest income group as this change also included a stronger reduction in the energy price levy.

The levels of the German carbon price are likely too low to achieve the emission targets mandated by the EU (see Fig. 2). Yet, the reform has introduced carbon pricing, along with the requisite institutional infrastructure. Correcting prices, especially increasing them, is easier now than it was previously. After all, it is remarkable that despite several obstacles, a Pigouvian policy has made its way through parliament and into political practice. Distributional concerns have been taken into account and accompanying measures implemented. Fragmented responsibilities among ministries have been overcome by means of an unofficial climate cabinet, with representatives of the ministries for the environment, finance, economy, agriculture and transport and infrastructure meeting regularly. Environmental groups, most notably the *Fridays for Future* movement, strongly advocated for carbon pricing — far from condemning the Pigouvian policy to be morally repugnant.

While there are reasons for calling the German case a success story of mitigation policies, the policy package remains imperfect. In addition to the sub optimally low carbon price in the German heating and transport sectors, the general problem of sector-specific policies remains unresolved. Effective carbon prices, including, e.g., pre-existing energy taxes, range from around 30 €/tCO_2_ in the electricity and non-electricity sectors, to more than 300 €/tCO_2_ in the transport sector (OECD 2018). The question as to how to cushion the social effects of energy price increases when carbon prices rise further and redistribution through existing channels becomes increasingly difficult is one of the future challenges that stand in need of resolution.

### Act 2: Towards a comprehensive EU carbon price

The sectoral fragmentation that plagues the German effort to decarbonize the economy is indicative of the even greater problem of sectoral fragmentation at the EU level. This is, however, not the only challenge European climate policy faces. The operation of the EU ETS is being disrupted by price volatility, policy-makers’ inability to commit and waterbed effects^6^ of national policies that interact with the trading scheme. Yet, the adoption of the European Green Deal might be an opportunity for EU climate policy to turn the tables. As we edge ever closer to a more comprehensive EU ETS, we may well be witnessing a Pigouvian moment right at the centennial of Pigou’s *The Economics of Welfare*. In what follows, we will critically review the shortcomings of the EU ETS and, more generally, European climate policy. Finally, we will offer some suggestions on how to redress these shortcomings.

The most obvious inefficiency in the EU’s climate policy program is its high degree of sectoral fragmentation. By design, the EU ETS covers only emitting entities above a certain threshold. Small installations, vehicles as well as buildings fall through the cracks. Thus, the trading scheme effectively covers only the industry and power sectors. The main instrument in the transport sector is a set of vehicle standards aimed at reducing emissions. Further, in the agriculture sector, the EU wide common agricultural policy (CAP) could provide incentives to reduce emissions through green subsidy schemes and programs. Unfortunately, the CAP is dominated by an income transfer motive rather than by environmental objectives. Emission targets in non-ETS sectors have to be met by member states through domestic policies. As elaborated in the preceding section, the German carbon price was a response to the EU Effort Sharing Regulation.

Aside from sectoral policies, the EU energy directive requires minimum energy taxes for various energy types that differ substantially when measured in carbon-content (Edenhofer et al. 2020), ranging from less than 5€/tCO_2_ for coal up to almost 180 €/tCO_2_ for gasoline.

There is growing pressure to reform the system of overlapping policies, which creates increasingly diverging incentives to decarbonize sectors, also implying a divergence in implicit or explicit carbon prices.^7^ The pressure will increase further as more ambitious mitigation targets are considered, which will tighten the EU 2030 reduction goal from 40% (compared to 1990 levels) to at least 55%. The EU’s Green Deal comprises an expansion of the EU ETS to include further sectors. Hence, the German national ETS could ultimately be integrated in the EU ETS, implying a harmonization of carbon prices across sectors and countries.

Further reform pressure in the EU ETS arises due to the waterbed effects with national policies, volatility of carbon prices and the inability to make credible commitments. The latter two problems are intertwined. Volatile allowance prices might reduce investment incentives and create additional economic costs (Nordhaus 2007), thus undermining the credibility of the cap-and-trade system (Flachsland et al. 2020). On the flip side, a large source of price volatility is associated with expectations and speculation about political decisions rather than changes in economic fundamentals (Koch et al. 2016). Also, the EU ETS reduction factor of 2.2% is only fixed until the end of Phase 4, that is, until 2030. This leaves the future development of the cap uncertain, which further impedes investments.

The introduction of the market stability reserve has helped to increase allowance prices recently—but the stability reserve also increased complexity in carbon price formation (Pahle et al. 2020). It therefore remains questionable whether the reserve can reduce price volatility (Perino and Willner 2016). A key alternative is to adopt a price collar like other ETS (as, for example in California or in Germany), thereby explicitly stabilizing price expectations and, concomitantly, reducing price volatility. Another advantage of a price collar is that unilateral policies might not be completely eliminated by the waterbed effect—in particular, when the minimum price becomes binding.

The European Green Deal will likely change the incentive structure of the European Commission and the EU member states. One particularly important element of the Green Deal is the proposal to allocate 20% of EU ETS auction revenues to the EU budget. As long as auction revenues were under the control of the EU member states, sectoral expansion of the EU ETS and the introduction of a minimum price would have been in their interest because it would have increased their auction revenues. However, when large parts of these revenues are channeled to the EU level, member states might be less interested in an efficiency-improving reform of the EU ETS. Additionally, the newly tightened EU emission target provides an incentive either to include the transport, heating and building sectors into the EU ETS or, at least, to create a second emissions trading scheme for these sectors.

However, the EU needs additional funding to repay debt caused by the recent COVID-19 crisis. EU member states might accept the proposal to allocate one fifth of the auction revenues to the EU if other contributions of the EU member states (e.g. GNI contributions) are reduced. It is worth noting that the declining tax base makes carbon pricing attractive, even for member states that are skeptical of funding the EU budget with new resources: These revenues provide sufficient funding to repay the EU's coronavirus borrowing and effectively imply a sunset clause as the tax base will eventually diminish. Additionally, climate policy can be perceived as a genuine European public good financed by the revenues from the EU emissions trading system (Fuest and Pisani-Ferry 2020). To conclude, the EU Green Deal has the potential to create a Pigouvian moment within the EU.

### Act 3: The role of carbon pricing in enhancing international cooperation

Climate policy in Germany, in the EU and in several other countries is advancing. In a recent video address to the United Nations General Assembly, president Xi Jinping announced China’s pledge to become carbon neutral by 2060. However, we are still far from bending the curve of global GHG emissions. Most notably, the continued use of coal in many developing economies is a large burden on the remaining global carbon budget implied by the Paris Agreement. To tackle the problem of the international renaissance of coal, international negotiations are necessary. In particular, they should focus on increasing domestic carbon minimum prices in combination with conditional transfers that help to overcome free riding and bring down the high cost of capital for renewable energy. Such a paradigm shift is a *conditio sine qua non* for the achievement of the Paris target.

A recent calculation by Edenhofer et al. (2018) suggests that achieving the 1.5 °C target is highly compromised by the already committed emissions of existing, under construction and planned coal-fired power plants across the world. It is worth noting that coal is highly attractive for developing and emerging economies as investing in renewable energy, instead of coal, entails much higher up-front capital costs. Therefore, the relatively high interest rates in these countries reduce the incentives for domestic green investments significantly (Best 2017; Hirth and Steckel 2016). The implication for carbon pricing in developing countries is then that the high capital costs lead to relatively ineffective carbon pricing, as measured by its ability to decarbonize the energy sector. Loans at preferential interest rates for low-carbon investments could therefore increase the penetrative power of carbon pricing substantially.

But, aside from financing low-carbon investments in countries with high capital costs, climate policy has been paralyzed by international free-rider problems and the absence of mechanisms to sanction free-riding and incentivize cooperation. While the Paris Agreement was celebrated as a success of the international community, critics argue that the agreement constitutes a Nash equilibrium with respect to the national emission reduction pledges. While the global temperature target is ambitious, the agreement lacks any strong enforcement mechanisms.

To overcome free-rider problems, a paradigm shift is necessary that moves away from quantity targets to carbon prices as a focal point of international negotiations (Cramton et al. 2015; P. Cramton et al. 2017a, b). Quantity targets are plagued by various problems, including the difficulty to measure the ‘effort’ of a country, which depends on a counterfactual baseline and (hardly verifiable) mitigation costs. Focusing on carbon pricing, instead, provides a clear long-term perspective for climate policy (a globally uniform carbon price that induces efficient mitigation and avoids carbon leakage), where temporary price differentials can be tolerated. The paradigm shift from quantities to prices also implies an overhaul of international climate finance—which has aimed at reducing emissions (quantities) on a project base. Within this approach, large financial flows have been generated to offset emissions, e.g., by afforestation projects in developing countries. Yet, the “additionality” of these projects—to what extent they have reduced emissions compared to the counterfactual emissions—has been highly disputed. Rather than financing mitigation projects, an international climate fund should support governments by helping them introduce or increase domestic carbon prices (Kornek and Edenhofer 2020). This mechanism promises to be more effective in reducing emissions abroad than project-based climate finance—and it helps to increase carbon prices and reduce price differentials over time.

### Progress made, lessons learned

We trust that the reader of the above three-act drama has developed an appreciation of the enormous success of economic policies based on Pigou’s fundamental analysis. In a rather unlikely reversal of political fortune, carbon prices have emerged in several countries around the globe. Their implementation might be fraught with inefficiencies, their scope might be too narrow and their level too low. But the outside observer of the whole process, at all different levels, has had a highly instructive opportunity to learn about the stepping stones for overcoming the barriers mentioned in Sect. 2. In the following, we examine each barrier and show how they have been dealt with in Germany and by the EU. In presenting the lessons learned, we hope to distill guidelines for the future of carbon pricing and its role in avoiding dangerous climate change (see Table 1).

**Table 1:** Barriers and solutions in Germany and the EU

Problem	Stepping stones toward a solution		Further recommendations
	In Germany	In the EU	
Uncertainty about costs and benefits	Price collar with minimum and maximum prices	Not yet addressed; price collar for EU-ETS is a reform option	Consider rules-based approaches to adjust carbon prices and the social cost of carbon
Unclear relation between CEA and CBA	Focus on EU-based quantity targets	Quantity targets (EU’s NDC) as vision resulting from societal discourse on risk management	Frequent updating of mitigation costs and damages; exploring the optimality of policies with different social welfare functions
Distributional concerns	No per-capita recycling, but reduction of regressive energy taxes	Effort Sharing Regulation with differentiated efforts; future: allocation of EU ETS revenues to solve distributional conflicts	Need for more research on horizontal equity, volatility of carbon pricing revenues and intertemporal allocation of revenues and debts, in particular w.r.t. carbon dioxide removal
Inefficient sector-specific policies	"Climate Cabinet "—instead of fragmented responsibilities of ministries	Incremental integration of EU ETS and non-EU-ETS into one common carbon market	Pursue efforts to mainstream climate change in overall policy-making (e.g., via Coalition of Finance Ministers and similar approaches); consider establishment of inter-agency institution for coordinating trade-offs and synergies between ministries and government divisions
Unstable tax base	Use of revenues for green spending, and temporary transfers rather than income tax cuts	Use revenues for financing ‘projects’ (COVID recovery)	Consider lump-sum redistribution of carbon tax revenues, as e.g., in Switzerland
Commitment device	The EU Effort Sharing Regulation; in future also the EU Green Deal	Still missing; but changes in policies require qualified majority or consensus among member states	Delegation of power to an independent carbon bank could be preferable under certain conditions
Repugnant markets	Price floor mimics direct pricing	Future minimum price could mimic direct pricing	Consider intelligent design to communicate carbon prices, e.g., via framing and labeling. Mainstream economic discussions about climate change in public debates
Lack of trust in government	Public debate and scientific assessment about different policies and their impact on emissions, distribution and costs	The EU Green Deal providing a genuine European public good financed by auction revenues	Need for joint learning process among scientists, policy-makers and citizens about different perceptions of fairness, trade-offs and functioning of specific policy measures
International cooperation	-	Explicit and implicit transfers to member states	Link climate finance and international transfers to the introduction of increasing carbon prices in other countries

Uncertainty about mitigation costs and benefits (social cost of carbon) is met by setting quantity targets for emission reductions, while complementing them with minimum and maximum prices. At the EU level, the targets are determined by the EU’s commitments, as enshrined in the Paris Agreement and the aim of the global community to avoid ‘dangerous’ climate change. Germany, then, has derived its pricing policies from the quota the EU has allocated to Germany.

Distributional concerns have been mostly dealt with at the national level. German policy-makers decided against the much-discussed tax-and-dividend approach, instead opting for reducing regressive energy taxes and large green spending programs.

There are reasons to expect that the sectoral fragmentation at the EU level will be ameliorated by an incremental integration of the current non-ETS sectors into the trading scheme. The German case is particularly interesting as the government has established a coordinating institution at the highest level of the executive branch. A so-called “Climate Cabinet” was formed that convened all ministries with responsibilities for national climate policy. This facilitated coordination of policies across sectoral boundaries. Internationally, a Coalition of Finance Ministers for Climate Action has emerged, which has committed itself to the so-called Helsinki Principles, one of which states that this group is to “work towards measures that result in effective carbon pricing”. This could also improve intra-governmental coordination, especially between the ministries of finance and of the environment.

The problem of the unstable tax base has, thus far, been addressed by earmarking the revenues for specific projects or temporary expenses, special-purpose funds or simple lump-sum transfers to citizens. Examples include the national Swiss carbon price, California’s Greenhouse Gas Reduction Fund, Chile’s Economic and Social Stabilization Fund and the revenue volatility management that France has implemented to deal with EU ETS auction revenues (World Bank 2019).

The lack of trust in government, in general, and in carbon pricing, in particular, remains an enormous challenge for economists, other experts, and the interaction between the latter and policy-makers. Here, substantial efforts are needed to start a joint learning process among scientists, policy-makers and citizens about different perceptions of fairness, trade-offs and functioning of specific policy measures (Kowarsch et al. 2016). Deliberative learning processes could help to improve the outcome of government processes and increase trust in government decisions. Empirically, there is a significant positive correlation between trust in governments and the quality of institutions, on the one hand, and carbon price levels, on the other (Klenert et al. 2018; Levi et al. 2020).

On the problem of repugnant markets, recent research on financial incentives and moral behavior suggests that direct pricing through taxes is better at tapping into moral behavior and intrinsic motives to reduce emissions than indirect pricing through emissions trading. In a recent experiment, Ockenfels et al. (2020) have shown that there is a fundamental difference between direct pricing and indirect pricing through emissions trading. While direct pricing increases voluntary abatement, indirect pricing reduces the latter. Market participants understand that voluntary abatement in indirect pricing schemes lowers the market price and therefore the incentive to abate. The experiment, thus, shows that a stable price incentivizes voluntary abatement.

While these considerations suggest that carbon taxes are better able to mobilize intrinsic moral behavior than emissions trading, labeling a Pigouvian price as ‘tax’ reduces public support significantly (Kallbekken et al. 2011). This phenomenon has been coined tax aversion (McCaffery and Baron 2003): Just by calling a specific instrument a tax, people tend to have negative association compared to other labeling. Labeling Pigouvian taxes as ‘fees’ or ‘charges’ has therefore been suggested to increase support (Baranzini and Carattini 2017).

Because of the European Commission’s power to use financial sanctions, the EU climate policy constitutes a strong commitment device for national climate policies. Nevertheless, credibility of financial sanctions might be weakened if national governments expect also many other governments to not comply with a specific directive. Because of consensus principles in many areas, the EU climate policy exhibits large inertia, impeding a flexible adjustment of climate policy to new information of technology costs, climate damages or climate policies in other countries.

## Remaining research gaps and research agenda

While some progress on carbon pricing has been made, there remain some fundamental challenges and significant gaps in our understanding of the theoretical underpinnings of Pigouvian pricing. Here, we will discuss the three areas that are most pertinent to public policy and, as such, merit closer attention: Macroeconomic stability and implications of the Covid-19 pandemic; commitment and credibility of climate policy; carbon pricing revenues and distribution.

### Macroeconomic stability and implications of the Covid-19 pandemic

The economic downturn caused by measures to contain the Covid-19 pandemic has demonstrated the momentous nature of global economic shocks. GHG emissions have fallen significantly. Unfortunately, this temporary, lockdown-induced decline only took us to the level of emissions produced in 2006 (Le Quéré et al. 2020)—and emissions have, historically, quickly started rising after crises (Peters et al. 2012). More importantly, the shock of the pandemic highlights the vulnerability of financial markets to tail events (Schnabel 2020).

While carbon pricing dominates the debate about climate policy instruments, finance experts increasingly argue for systematically assessing other options to enhance mitigation efforts and to incorporate risks of climate impacts as well as mitigation policies into broader macroeconomic policies (Campiglio 2016; IMF 2019b; Schnabel 2020). In this respect, there are three different types of macroeconomic policies: Fiscal policy, financial policy and monetary policy. Optimal carbon pricing, a fiscal policy tool, is a necessary condition for the market failure caused by GHG emissions to be corrected. Additionally, governments might have to increase spending for the energy transition, e.g., related to public goods like infrastructure or innovation.

However, fiscal policy likely has to be complemented by financial and monetary policy tools in order to facilitate private finance flows directed at low carbon investments and to mitigate systemic risks associated with climate policy. These systemic risks arise due to tail events related to physical climate impacts, green technology breakthroughs or bursting fossil asset price bubbles (Bolton et al. 2020). Financial policy can help redress the underpricing and the lack of transparency of climate risks. It can also address short-term biases, improve governance frameworks of financial institutions, support the development of green financial securities and promote climate finance.

Carbon pricing as well as climate impacts might have strong impacts on relative prices and, thus, inflation levels (Diluiso et al. 2020). Therefore, they strongly interact with Central Banks’ mandate of price stability. Optimal financial portfolios are often determined by backward-looking assessments of returns. Climate change and climate policy require forward-looking models and scenario approaches for managing financial portfolios—by financial investors as well as central banks (Bolton et al. 2020). Moreover, green quantitative easing and collateral frameworks could be established and combined with appropriate credit allocation policies to avoid investments into fossil-based capital stocks (IMF 2019b). The ECB, for example, is taking first steps to improve disclosure requirements and to reduce informational inefficiencies, but may also consider excluding bonds that conflict with the decarbonization objectives of the EU—a step that will be discussed by the ECB Governing Council at the next monetary policy strategy review (Schnabel 2020). The literature offers little insights into frameworks for discussing the most effective policy mix of these tools. The coordination of instruments seems, however, very important, in particular owing to the unprecedented scale of climate change. Moreover, there is an important concern that central banks might overstep their mandate when engaging actively in climate policy, filling the fiscal and environmental policy gaps arising from insufficient government action (EPP Group in the European Parliament 2020). In particular, central banks might not be able to achieve multiple objectives when a clear mechanism to evaluate trade-offs is missing and banks lack the requisite tools for effectively addressing each objective (Dikau and Volz 2020).

### Commitment and credibility of climate policy

Improving commitment and credibility in climate policy and carbon pricing remains an important challenge. Under certain circumstances, it is preferable to delegate decisions and responsibilities to a technocratic body in order to remove decisions from direct political influence. Economic theory has provided some general insights, which can be applied to climate policy (Benassy-Quere et al. 2019). Based on these considerations, an independent European carbon bank might be a preferable option to strengthen commitment if the following conditions are fulfilled:

1. *The emission reductions pathway or the carbon budget is well-defined by legitimate democratic institutions*. This implies that the ultimate goals of climate policy are determined by democratically elected governments.
2. *The performance criteria for implementing policy instruments are well-understood*. The carbon bank administers the auctioning process of permits, implements a minimum price and manages the inclusion of all relevant sectors. Additionally, the carbon bank provides subsidies to negative emission technologies. It also has to approve and regulate new negative emission technologies according to politically determined criteria, monitor relevant secondary market failures that impede the effectiveness of carbon pricing and assess whether investments into fossil and carbon-free technologies are consistent with the overall long-term climate goal.
3. *The carbon bank acts independently of any revenue objectives and is independent from lobbying by firms or national governments*, as this increases the risk of time-inconsistent carbon pricing (Kalkuhl et al. 2020). The institutional design has to ensure this independence.
4. *The carbon bank does not consider distributional effects between member states or among the income distribution as part of their mandate to avoid following multiple objectives*. National governments are responsible for addressing distributional effects within their jurisdictions via their national tax systems. Distributional impacts across countries, in particular concerning the revenues from carbon prices (e.g., due to adjusting the cap or the minimum price), should be governed by pre-defined effort sharing principles, which member states agreed upon. The intergenerational distribution is determined by an efficient dynamic use of the carbon budget and the net negative emission technologies. Both have to be adjusted when new scientific information about climate impacts and mitigation costs become available. Ideally, the adjustment is based on pre-defined rules.
5. *A minimum price within the EU emissions trading scheme* might allow considering idiosyncratic preferences of the EU member states on climate mitigation. Complementary policies of EU member states are less distortionary compared to a trading scheme without minimum prices.

It is debatable whether an institution, like an independent carbon bank, can be designed according to the above principles, thereby reducing the vulnerability to time inconsistency. Two fundamental research questions have to be answered: First, what is the optimal degree of ‘delegation’ with regard to the unavoidable trade-off between investment security and democratic principles to adjust policies? Second, what are optimal rules for setting carbon prices or price collars? In particular, the optimal rules must determine how marginal costs and benefits are measured—and how domestic carbon prices are strategically linked to carbon prices in other countries to enhance international coordination.

### Carbon pricing revenues and distribution

Finally, more attention should be paid to the question of integrating carbon pricing within the broader fiscal systems of (national) governments. In particular, there is a need for more research on questions of horizontal equity of carbon pricing, the volatility of carbon pricing revenues and intertemporal allocation of revenues and debts associated with Pigouvian policies.

There is already some research on the public economics of climate change that examines the fiscal properties of carbon pricing (de Mooij et al. 2012; Edenhofer et al. 2017; Jones et al. 2013; Siegmeier et al. 2018). Pigouvian taxation has also been studied in optimal taxation frameworks (for an excellent overview, see Aronsson and Sjögren 2018), where most attention has been given to the double dividend hypothesis (Goulder et al. 2016) and distributional effects with respect to different incomes (e.g., Jacobs and van der Ploeg 2019).

However, most models used to study the role of carbon pricing for the optimal tax portfolio are limited to income distributions along the vertical dimension, that is, between different income groups. Little is known about horizontal equity between heterogeneous households of the same income decile (Atkinson and Stiglitz 1976; Fischer and Pizer 2019), even though empirical studies have demonstrated that distributional impacts of climate policy within income groups show the largest variation (Cronin et al. 2018; Pizer and Sexton 2019; Poterba 1991; Rausch et al. 2011).

Moreover, a finance ministry seeking to integrate carbon pricing within the general system of public finance will very likely have to deal with the problem of managing an uncertain and potentially volatile revenue stream. This is due to (a) the unknown impact of carbon pricing on the economy (e.g., the speed of its decarbonization) and (b) the imperfect political process of implementing carbon pricing (e.g., how quickly effective price levels and full sectoral coverage can be reached). Managing such uncertainties sustainably remains an open research question.

Finally, to reach ambitious mitigation targets, mitigation scenarios show the importance of carbon dioxide removal (CDR) technologies (Strefler et al. 2018). In contrast to the harmful over-production of GHG emissions by private agents, such CDR technologies are subject to a harmful under-provision by private markets. Hence, theory tells us that Pigouvian subsidies are required. This raises several questions about optimal fiscal policy. For example, it is unclear how to generate the necessary public funds to finance such subsidies. It is also unclear how to achieve optimal intertemporal smoothing of the streams of public income and public debt associated with Pigouvian policies.

## Conclusions

The challenges of Pigouvian taxation should not be dismissed too easily. There are, indeed, thorny problems—both of a political and theoretical nature—that cast doubt on the political feasibility of Pigouvian taxation: uncertainties about marginal social benefits; moral objections to putting a price tag on environmental goods and services; coordination and responsibility problems within governments; a lack of ability to commit to Pigouvian pricing principles; a lack of trust in effectiveness and fairness of a Pigouvian tax, even when supplemented with sophisticated compensation schemes; the diminishing tax base demanding frequent adjustments in the government budget; and lastly, international cooperation on climate policy that has been notoriously hard to achieve.

But an excessive focus on the challenges of Pigouvian taxation unjustly disregards the growing political successes of carbon pricing. Today, 100 years after the publication of Pigou’s magnum opus, we see remarkable success stories, even under second-best conditions. There is a Pigouvian moment within the EU, as evidenced by its ambitious climate targets. The momentum is, however, still fragile and needs to be solidified as part of the next reform steps.

The fragility of Pigou’s success becomes apparent when considering recent criticisms of carbon pricing. The latter charge Pigouvian taxation with either being ‘politically infeasible’ (Bell 2018; Cullenward and Victor 2020; Heal 2020) or, at best, achieving marginal improvements, while failing to implement an all-encompassing transformation of our societies to carbon neutrality (Rosenbloom et al. 2020). Nevertheless, such critiques often remain silent when it comes to the economic costs and distributional effects of alternative paradigms. Given that there is no clear consensus on the more general drivers of ‘political feasibility’, it also remains unclear how to evaluate the political feasibility of alternative paradigms rigorously. As the reflections on the double dividend and the distributional effects have shown, it is useful to broaden prevailing economic models by endogenizing aspects that had been considered an exogenous constraint. This approach of considering multiple distortions actually corresponds to the Pigouvian principle of determining the level of tax that maximizes welfare.

A key political and institutional challenge consists in enhancing state capacity to assess and monitor market failures and externalities in a rigorous and comprehensive way. Governments devote much effort to collecting data on economic indicators, like GDP, employment, inflation, prices and expenditures. Yet, governments often lack a basic understanding of the size of market failures and externalities that deprive their citizens of their economic potential. An institution, tasked with rigorously and regularly assessing externalities, is a pre-condition for increasing the political feasibility of Pigouvian pricing. This is also important with respect to identifying related market failures that dilute the power of Pigouvian pricing (e.g., principal-agent or information problems in the building sector) as well as responding swiftly to new externalities that might be triggered by addressing other externalities (e.g., biodiversity losses due to increased biomass use under high carbon prices).

We conclude our tribute to Arthur Cecil Pigou with an invitation to follow his lead by tying rigorous economic theory to the pressing issues of our time and by making it relevant to actual modern societies. Economists can help significantly to enhance the implementation of the Pigouvian legacy by addressing research gaps related to the macroeconomic dimensions of Pigouvian taxation, by finding ways of enhancing commitment as well as setting up compensation schemes that are consistent with various normative principles. And they should rigorously compare Pigouvian approaches with alternative paradigms that seem to be politically less demanding. The latter is indispensable to enable a deliberative public discourse on efficient and fair environmental policy—and to increase trust in economists, experts and advisors. These steps are vital if we are to follow Pigou’s example as a public intellectual, attuned to the subtleties of the academic literature, cognizant of political realities and passionate about helping our societies deal with economic, environmental and broader societal problems.

## Footnotes

1 Pigou citing this report shows that he did not only expatiate upon environmental problems within a theoretical framework, but that he also thought about ways of using empirical data to estimate environmental damages (Sandmo 2015).

2 See the IEA’s *fossil fuel subsidies data base*, https://www.iea.org/topics/energy-subsidies (accessed on November 12, 2020).

3 Among these are Milton Friedman, Joseph Stiglitz, Greg Mankiw, Bill Nordhaus, Lord Nicholas Stern and Pope Francis.

4 Additionally taking into account other taxes and levies not explicitly targeted at GHGs results in a higher percentage of emissions covered by *effective* carbon prices (see Fig. 1).

5 Ongoing research by van der Ploeg, Rezai and Tovar (IIPF conference, 2020) also suggests a theoretical efficiency argument against lump-sum rebates of carbon pricing revenues. Such a scheme would not take the labor supply reaction into account. The increase in consumer prices would effectively harm poorer households more if lump sum transfers are used compared to a reform of non-linear income taxes.

6 A waterbed effect can occur when there is an emissions trading scheme in a union of several sovereign jurisdictions and emission certificates can be traded freely across the jurisdictions’ borders, as, e.g., in the EU. If one of the members of the union unilaterally introduces an additional policy that effectively reduces this member’s emissions, demand for emission certificates falls in this jurisdiction. Other jurisdictions can now buy the newly available certificates. With the top-level policy in place, reducing emissions in one jurisdiction merely leads to a relocation of emissions but not a reduction beyond the cap mandated by the top-level policy. The relocation of emissions without total reduction is akin to pressing down on one side of a waterbed leading to water just flowing to the other side without reducing the total amount of water in the bed.

7 The transport sector is subject to both high taxes on fossil energy and efficiency standards, which are tightened increasingly. In the building sector, there is only a set of EU minimum taxes, which are quite low, i.e., mostly below 10 €/tCO_2_ (Edenhofer et al., 2020). CO_2_ and other GHG emissions, caused by the agriculture sector, are not taxed at all.
